# Porcine cysticercosis in slaughtered pigs and factors related to *Taenia solium* transmission amongst abattoir workers in Ibadan, Nigeria

**DOI:** 10.11604/pamj.2019.32.145.10695

**Published:** 2019-03-26

**Authors:** Hezekiah Kehinde Adesokan, Fiyinfoluwa Adedolapo Adeoye

**Affiliations:** 1 Department of Veterinary Public Health and Preventive Medicine, University of Ibadan, Ibadan, Nigeria

**Keywords:** Cysticercosis, human health, Nigeria, pigs, surveillance, transmission

## Abstract

**Introduction:**

porcine cysticercosis is under-reported particularly in Nigeria, despite the reportedly high prevalence of epilepsy and associated life-threatening health implications. This study was aimed at determining the prevalence of porcine cysticercosis and factors related to *Taenia solium* transmission to humans.

**Methods:**

slaughtered pigs at a major abattoir, south-western Nigeria were randomly inspected and questionnaire was administered to pig workers/consumers while the data were analysed using Stata 12.0.

**Results:**

a 4.4% (11/250) prevalence of porcine cysticercosis was obtained; the age, breed, sex and body conditions of pigs were not significant for infection (p < 0.05). Further, none (0.0%) of the respondents knew that *T. solium* could cause epilepsy in man and 39.5% often defaecated on neighbouring open fields and farmlands. Respondents purchasing pork from home slaughter were about four and ten times less likely to demonstrate good knowledge (OR = 0.24; 95% CI: 0.08-0.74) and practice (OR = 0.10; 95% CI: 0.05-0.22) than those purchasing from abattoir. Moreover, those lacking toilet facility were about four and five times less likely to demonstrate good knowledge (OR = 0.24; 95% CI: 0.07-0.86) and practice (OR = 0.19; 95% CI: 0.08-0.45) than those who had it. Other factors associated with practices related to *T. solium* transmission included age (p = 0.000), sex (p = 0.000) and duration (p = 0.003).

**Conclusion:**

the increased odds of poor knowledge and practices related to *Taenia solium* transmission especially among respondents purchasing home slaughter pork and lacking toilet facility provides insights into the parasite epidemiology. Above findings are important in lowering the infection prevalence in pigs and humans in this endemic area.

## Introduction

Porcine cysticercosis, caused by *Taenia solium* is a global emerging socio-economic and public health problem [1]. Domesticated pigs are the natural host of the parasite and play a major role in the transmission cycle considering their close proximity to humans and the increasing pig keeping and pork consumption in many developing countries [2]. Available reports indicated that pigs are a potential protein deficit gap-filler, investment alternative and source of additional income to humans especially women [3, 4]. In fact, pork is the most widely eaten meat in the world accounting for over 36% of the world meat intake [5]. However, *T. solium* cysticercosis is a limiting factor to porcine husbandry especially in many developing countries of Latin America, Africa and Asia [6, 7]. Though pigs could have massive infections, the disease is rarely associated with symptoms since most pigs are slaughtered before nine months of age, a time too short for the cysts to reach degenerative stage [8]. Nigeria has an estimated pig population of 6.54 million pigs (http://www.fao.org/ag/againfo/resources/en/glw/GLW_dens.html ) characterized with semi-intensive system of management with the prevalence rate of porcine cysticercosis ranging between 6.25% in the North and 20.5% in some other parts [9, 10]. Most pigs in the country are raised in rural settings where there are close interactions between pigs and humans. Moreover, some of the pigs are purchased and brought to abattoirs at urban centres for slaughter while others are slaughtered at unapproved places especially in the rural areas, putting prospective buyers at risk of *T. solium* infections.

*Taenia solium* infection is related to poverty, absence of latrines as well as free access by roaming pigs to human faeces deposited indiscriminately on heaps and farmlands [11]. The spread of infection could also be enhanced by poor hygiene, inadequate sanitation and the use of untreated or partially treated wastewater in agriculture [12]. It is estimated that over 10 million people are exposed to this larval worm which frequently causes neurocysticercosis and epileptic seizures among those affected [13]. During the last decade, more attention has been given to this zoonosis in sub-Saharan Africa because of the growing recognition of the importance of neurocysticercosis in epilepsy [14]. Neurocysticercosis is the most common central nervous system parasite infection. It is an important public health problem in developing countries particularly in Africa as well as Asia and the Americas [15]. A significant number of new cases have been reported particularly in industrialized countries following increased immigration from endemic areas [16, 17]. In Nigeria and other developing countries, studies have consistently found higher prevalence of epilepsy in rural than in urban areas [18-20]. In addition, a previous study showed that consumption of pork infected with *T. solium* could predispose people to epilepsy and it's a main cause of acquired epilepsy in endemic low income countries including sub-Saharan Africa [21]. This is further supported by a meta-analysis of studies conducted in Africa which found a significant link between cysticercosis infestation and the occurrence of epilepsy [22]. Despite the high numbers of *Taenia* spp. carriers (8.6%) [9] and the high prevalence of epileptics in Nigeria (37 per 1000) [23], data on knowledge and practices of pig workers/consumers in relation to *T. solium* transmission to humans are limited. This study was therefore aimed at determining the current prevalence of *T. solium* cysticercosis in pigs brought for slaughter from rural settings as well as potential risk factors related to its transmission to humans in Ibadan, south-western Nigeria.

## Methods

**Study design, site and sample size determination**: this cross sectional study was carried out at the Bodija Municipal Abattoir in Ibadan, south-western Nigeria. This is the biggest abattoir in the area which supplies bulk of the meat consumed by the residents of Ibadan metropolis and its surrounding environment. Ibadan is the capital of Oyo State in South-Western Nigeria and lies between latitude 7^0^32^1^N and longitude 3^0^54^1^E, with a population of 2 893 137 in 2010 [24]. The abattoir was chosen as the population of animals slaughtered there represents more than 60% of all food animals slaughtered in the area. There is a growing demand for pork in the area considering the analysis of animal product consumption pattern among households in south-western Nigeria which attributed 4.6% to pork when compared to 4.3%, 4.13%, 3.4% and 1.05% for goat meat, turkey, bush meat and mutton, respectively [25]. The sample size of pigs that were sampled for the study was computed using the formula:

$$n=\frac{Z\alpha^{2}\text{pq}}{L^{2}}$$

…[26] where, n is the required sample size, Zα = 1.96 is the standard normal deviate at 5% level of significance, p is the estimated prevalence, q = 1-p, and L is the precision of the estimate. Setting p = 0.0625 [10] and L at 5%, the required minimum sample size was 90 pigs. However, we sampled a total of 250 pigs considering the volume of pigs slaughtered per day and in order to minimize the possibility of chances. In the same vein, from an average of 258 slaughterhouse workers who met the inclusion criteria of either working directly with pigs in the abattoir, had pigs at home or eat pork, a total of 200 respondents were willing to participate in the study. Those who had neither handled pigs nor ate pork were excluded from the study.

**Animal sampling, questionnaire administration and data analysis:** prior to slaughter, pigs were randomly selected and given identification numbers and data on the age, breed, sex as well as body condition scores were taken with emaciated ones scored as poor and apparently healthy, well fleshed pigs as good. After slaughter, the selected pigs were inspected using palpation and deep incision techniques during routine meat inspection. The masseter muscles, heart, the gracilis muscles and diaphragm were visually inspected, palpated and incised while the oesophagus, liver, lungs, spleen, stomach, subcutaneous fat and kidney were visually inspected for the presence of cysts [27]. The identified animals and their respective organs were examined strictly separately to avoid mixing the organs. The cysts were identified as being alive based on fluid-filled translucence and invaginated visible scolices; and dead as bluish green caseous masses [9]. A structured questionnaire was designed and administered personally by the authors to willing abattoir workers who met the inclusion criteria on the study site. The content validation of the questionnaire was done by cross-reference (Cronbach's alpha = 0.7834). A pre-test of the questionnaire was initially carried out after which some of the questions were modified to improve clarity.

The questionnaire included three parts to respectively document the respondents' socio-demographic data including age, sex, duration spent as pig workers/consumers, etc; ascertain their knowledge level on porcine cysticercosis and its transmission as well as determine behavioural practices that could enhance human infection. The knowledge section consisted of seven questions while the practice assessment had nine questions. Respondents were told to choose an option from a list of options for each question in the different sections. Each correct response was scored one and incorrect response zero and the scores were converted to 100% and then classified as poor (marks below 50%) and good (marks above 50%). The data obtained were analyzed using Stata 12.0 (StataCorp LP, Texas, USA). Prevalence of cysts was recorded as percentage based on sex, age, breed and body conditions of pigs. Univariate analysis was first conducted on all variables from pig sampling and questionnaire administration using Chi-squared test statistic to determine potential variables for the logistic regression model. A multivariate unconditional logistic regression analysis was then performed on the variables that were statistically significant at 10% level. All tests were two-tailed and p-value of less than or equal to 5% were considered significant. The odds ratios were reported with their 95% confidence intervals (CI).

**Potential benefits and hazards, recruitment procedures, informed consent and data protection**: there was no risk associated with the respondents as the authors ensured that individual identities were not attached to the data obtained. Possible fears relating to envisaged apprehension about the plausible consequences of their poor practices for *T. solium* transmission were taken into consideration. They were encouraged to visit their doctors for regular de-worming and medical check-ups. The respondents became better enlightened on *T. solium* transmission; hence limited infection spread to the society in general. Participation in the study was based on willingness after the purpose of the study had been explained to the potential participants. They were told they could withdraw their consent to participate in the process of the interview in the event that they felt the need to do so, without any attached penalty. Given the literacy level of most of the respondents, verbal consent was obtained from those who willingly chose to participate in the study. Data obtained from each respondent was handled with utmost confidentiality without divulging information from one respondent to the other. The authors labelled each questionnaire with use of codes so that no names or any other identities of the respondents could be linked to the data. This study had some limitations. One, the tongues of the pigs screened were not examined by deep incisions due to the uncooperative attitudes of the butchers as they had the erroneous belief that such incisions would lower the market value of this organ. Two, the respondents were not investigated for the presence of taeniid eggs despite the associated risk practices and poor knowledge. Doing this would have given further credence to the public health implications of our findings. We however, recommend that future research should investigate this aspect in order to establish the burden of this parasite in the occupationally exposed group.

## Results

**Animal sampling:** from a total of 250 pigs examined, 11 (4.4%) had cysticerci of *T. solium*. Based on variables studied, the young adult (7.5%), the boar (6.1%), the duroc breed (9.1%); and pigs with poor body scores (6.6%) were the most affected with no significant association with infection (p > 0.05) (Table 1). With respect to predilection sites, 10 (90.9%) cases were from subcutaneous fat and one (9.1%) from the liver.

**Table 1:** distribution of slaughtered pigs examined and infection status with respect to age, sex, breed and body conditions (N=250)

Variables	Categories	Number inspected	Number infected (%)	p-value
Age	Adult	210	8 (3.8)	0.39
	Young	40	3 (7.5)	
Sex	Female	152	5 (3.3)	0.34
	Male	98	6 (6.1)	
Breed	Large white	164	7 (4.3)	0.62
	Hampshire	57	3 (5.3)	
	Duroc	11	1 (9.1)	
	Cross breed	18	0 (0.0)	
Body condition	Poor	91	6 (6.6)	0.22
	Good	159	5 (3.1)	
Total		250	11 (4.4)	

**Questionnaire administration:** out of a total of 200 pig workers/consumers who participated in the study, 56.5% were between the age groups 41 years and above, 78.5% were male respondents, 41.5% had no formal education and 83% had been pig workers/consumers for over three years (Table 2). Moreover, 13% were not pork consumers while a high proportion (73.0%) of the respondents who consumed pork purchased it from home slaughter. Also, 39.5% of the respondents did not have toilet facility at home, but defaecated on open fields and farmlands around their areas (Table 2). The results show that 89.5% had never heard of porcine cysticercosis and only 17.5% had taken note of cysts in pig carcasses before. While 67.5% knew someone with epilepsy, none (0.0%) however; knew that porcine cysticercosis could cause epilepsy in humans. Again, none (0.0%) of them knew that humans could be infected with *T. solium* cysticercosis. In addition, none (0.0%) of the respondents knew that improper washing of hands after using the toilet as well as lack of regular deworming could facilitate human infection with *T. solium*.

**Table 2:** knowledge levels of respondents (n =200) in relation to *Taenia solium* cysticercosis and their socio-demographic characteristics as well as behavioural practices

Variables	Good N (%)	Poor N (%)	Total N (%)	Bivariate p-value	Logistic regression OR; 95% CI; *p*-value
**Age groups (in years)**					
< 20	1 (16.7)	5 (83.3)	6 (3.0)		REFERENCE
20 – 30	2 (5.4)	35 (94.6)	37 (18.5)	0.056	0.29; 0.02-3.76; 0.341
31 – 40	9 (20.5)	35 (79.5)	44 (22.0)		1.29; 0.13-12.43; 0.828
≥41	8 (7.1)	105 (92.9)	113 (56.5)		0.38; 0.04-3.67; 0.403
**Sex**					
Male	2 (1.3)	155 (98.7)	157 (78.5)	0.000	REFERENCE
Female	18 (41.9)	25 (58.1)	43 (21.5)		55.8; 12.20-255.31; 0.000
**Education status**					
No formal education	4 (4.8)	79 (95.2)	83 (41.5)		
Primary	10 (13.3)	65 (86.7)	75 (37.5)	0.119	NA*
Post-primary	6 (14.3)	36 (85.7)	42 (21.0)		
**Duration as pig workers/consumers (in years)**					
≤3	3 (8.8)	31 (91.2)	34 (17.0)	1.000	NA*
> 3	17 (10.2)	149 (89.8)	166 (83.0)		
**Pork consumption**					
Yes	14 (8.1)	160 (91.9)	174 (87.0)	0.029	0.29; 0.10-0.85; 0.023
No	6 (23.1)	20 (76.9)	26 (13.0)		REFERENCE
Purchased pork from(**N=174)					
Abattoir	8 (17.0)	39 (83.0)	47 (27.0)		REFERENCE
Home slaughter	6 (4.7)	121 (95.3)	127 (73.0)	0.008	0.24; 0.08-0.74; 0.013
**Toilet use at home**					
Toilet facility	17 (14.0)	104 (86.0)	121 (60.5)	0.018	REFERENCE
Open field/farmlands	3 (3.8)	76 (96.2)	79 (39.5)		0.24; 0.07-0.86; 0.027
Total	20 (10.0)	180 (90.0)	200 (100.0)		

* NA**:** not applicable as p-value at bivariate analysis is more than 10%

** The respondents (n=26) who were not pork consumers were excluded from the analysis

Of the 73.0% respondents who purchased pork from home slaughter, only 4.7% had good knowledge of porcine cysticercosis. This factor was significantly associated with the level of knowledge of the respondents regarding porcine cysticercosis (p = 0.008). Of the 39.5% who had no toilet facility at home, only 3.8% had good knowledge of porcine cysticercosis and this was significantly associated with knowledge level (p = 0.018). Those who purchased pork from home slaughter were about four times less likely to demonstrate good knowledge than those purchasing from the abattoir (OR = 0.24; 95% CI: 0.08-0.74; p = 0.013). Again, those who did not have toilet facility at home were about four times less likely to demonstrate good knowledge than those with toilet facility (OR = 0.24; 95% CI: 0.07-0.86; p = 0.027). In addition, knowledge level was significantly associated with sex (p = 0.000); the female respondents being about 56 times more likely to demonstrate good knowledge level than the male respondents (OR= 55.8; 95% CI: 12.20 - 255.31) (Table 2).

Moreover, none (0.0%) of the respondents who consumed pork ate it raw or uncooked, however; 5.5% consumed “suya” (undercooked meat type) made from pork. Although, the majority (92.5%) of the respondents indicated that they made use of toilets in the abattoir while at work, 39.5% defaecated on open fields or farmlands around their areas when at home for lack of toilet facility. While all (100.0%) indicated that they always washed their hands after using toilets, only 43.0% did proper hand washing using soap and water. Again, from 30.5% who indicated that they de-wormed regularly; 65.6% had not de-wormed within the last six months. In addition, out of the 73.0% respondents who purchased pork from home slaughter, only 11.8% had good practice of porcine cysticercosis, a factor which was significantly associated with their practice (p = 0.000). Of the 39.5% who had no toilet facility at home, only 8.9% demonstrated good practices regarding prevention of *T. solium* transmission and this was significantly associated with practice (p = 0.000) as well. Those who purchased pork from home slaughter were about 10 times less likely to demonstrate good practice than those who purchased from the abattoir (OR = 0.1; 95% CI: 0.05-0.22; p = 0.000). Again, those lacking toilet facility at home were about five times less likely to demonstrate good practice than those who had it (OR= 0.19; 95% CI: 0.08- 0.45; p = 0.000). In all, age (p = 0.000), sex (p = 0.000) and duration (p = 0.003) of respondents were significantly associated with their practice level. The female respondents were about 21 times more likely to demonstrate good practices than the male respondents (OR = 21.27; 95% CI: 9.23 - 49.04). Respondents who had been pig workers/consumers for more than three years were about four times less likely to demonstrate good practices (OR= 0.27; 95% CI: 0.12 - 0.58) than those with less than three years (Table 3).

**Table 3:** practice levels of respondents (n =200) in relation to *Taenia solium* transmission and their socio-demographic characteristics as well as behavioural practices

Variables	Good N (%)	Poor N (%)	Total N (%)	Bivariate *p*-value	Logistic regression OR; 95% CI; *p*-value
**Age groups (in years)**					
< 20	4 (66.7)	2 (33.3)	6 (3.0)		REFERENCE
20 – 30	14 (37.8)	23 (62.2)	37 (18.5)	0.005	0.30; 0.05-1.88; 0.201
31 – 40	6 (13.6)	38 (86.4)	44 (22.0)		0.08; 0.01-0.53; 0.009
≥41	24 (21.2)	89 (78.8)	113 (56.5)		0.13; 0.02-0.78; 0.025
**Sex**					
Male	17 (10.8)	140 (89.1)	157 (78.5)	0.000	REFERENCE
Female	31 (72.1)	12 (27.9)	43 (21.5)		21.27; 9.23-49.04; 0.000
**Education status**					
No formal education	17 (20.5)	66 (79.5)	83 (41.5)		
Primary	21 (28.0)	54 (72.0)	75 (37.5)	0.543	NA*
Post-primary	10 (23.8)	32 (76.2)	42 (21.0)		
**Duration as pig workers/consumers (in years)**					
≤3	16 (47.1)	18 (52.9)	34 (17.0)	0.001	REFERENCE
> 3	32 (19.3)	134 (80.7)	166 (83.0)		0.27; 0.12-0.58; 0.001
**Pork consumption**					
Yes	42 (24.1)	132 (75.9)	174 (87.0)	0.906	NA*
No	6 (23.1)	20 (76.9)	26 (13.0)		
Purchased pork from(**N=174)					
Abattoir	27 (57.4)	20 (42.6)	47 (27.0)		REFERENCE
Home slaughter	15 (11.8)	112 (87.2)	127 (73.0)	0.000	0.10; 0.05-0.22; 0.000
**Toilet use at home**					
Toilet facility	41 (33.9)	80 (66.1)	121 (60.5)	0.000	REFERENCE
Open field/farmlands	7 (8.9)	72 (91.1)	79 (39.5)		0.19; 0.08-0.45
Total	48 (24.0)	152 (76.0)	200 (100.0)		

* NA: not applicable as p-value at bivariate analysis is more than 10%

** The respondents (n=26) who were not pork consumers were excluded from the analysis

## Discussion

We report a 4.4% prevalence of porcine cysticercosis in slaughtered pigs in Ibadan, south-western Nigeria with attendant increased odds of poor knowledge and practices related to *T. solium* transmission among pig workers/consumers. We also document a high proportion of the respondents purchasing pork from home slaughter which lacked veterinary supervision and meat inspection. In addition, almost 40% lacked toilet facility at home and often defaecated on open fields and farmlands in such a porcine cysticercosis endemic area where pigs are mostly managed on semi-intensive system whereby pigs are allowed to roam freely in search of feed and then return later in the day. The prevalence of 4.4% of porcine cysticercosis obtained in this study is closer to a previously reported prevalence of 5.5% in southern Nigeria [7]. However, it is much lower than 13.5% [9] and 9.5% [28], respectively reported in eastern and northern Nigeria and 20.6% in Zambia [2]. This difference could be explained by the higher dog population in the present study area when compared with the much lower population in other areas particularly the Northern Nigeria. As reported, dog to human ratio is higher in Lagos (same region with the present study area) south-western Nigeria being 1:21 in contrast to 1:1 000 in the Moslem dominated parts of Northern Nigeria [29]. Since dogs have a more developed olfactory lobe, it is possible that they will locate the faeces more easily and faster than pigs, thereby reducing the chances of pigs getting infected during free ranging. Hence, the relative lower prevalence in this area of study with higher dog population. This assertion is supported by the previous reports [30, 31] which indicated that dogs are highly susceptible and become intermediate host and may be involved in the completion of the life cycle of the parasite. Furthermore, the results of this study revealed that age, sex and breeds of the pigs screened were not significantly associated with infectivity with *Cysticercus*. This observation is in line with the reports of previous workers [9, 12] which showed that age and sex did not have significant influence on infectivity of *Cysticercus* cysts since pigs were exposed to similar poor sanitary conditions and stress. Our observation is further supported by the report [7] showing no significant association between the sex of pigs and the infection. A similar finding was recorded in The Gambia and Senegal [32] where the age and sex of pigs screened were not significantly associated with occurrence of porcine cysticercosis. The pigs in this study area were exposed to similar conditions of semi-intensive system of management whereby they roamed about irrespective of age, sex or breed.

The presence of most of the cysts detected in this study in the subcutaneous fat is in agreement with a previous report which indicated that Cysticerci lodge anywhere in the body of the pig, most commonly in the muscle and subcutaneous fat [8]. This finding is however, in contrast with previous reports showing that cysts were most common in the shoulder and masseter muscles [33, 34]. The difference might be explained by the resistance often encountered from butchers by meat inspectors when attempting to carry out detailed post-mortem examination involving deep incision in most abattoirs in Nigeria. Hence, the possibility of missed cases might not be ruled out in this study. This notwithstanding, the prevalence portends a potential health threat considering free ranging of pigs in the area. Home slaughter of animals is common in most developing countries and unsuspecting buyers tend to patronise such places despite their poor knowledge about zoonoses. A report showed that home slaughtering of pigs with only limited or no inspection of the carcass/meat as well as frequent consumption of undercooked pork at local brew bars was a common practice in the southern highlands of Tanzania [35]. While the lack of regular meat inspection, particularly in unregistered slaughter premises, has the tendency to promote the sale and consumption of unwholesome pork products, a report showed that infected pork is usually sold at a decreased price at such illegal premises in Cameroon [36]. As seen in this study, 73.0% of pork consuming respondents purchased pork from home slaughter without veterinary supervision and meat inspection. This becomes worrisome when only 4.7% of these had good knowledge of porcine cysticercosis. Previous studies have established home slaughter as a major risk factor for *T. solium* transmission to humans since lack of meat inspection is critical in the transmission to humans [12] and exacerbates the cysticercosis-taeniasis complex. This observation is buttressed by the report which identified home slaughter of pigs without meat inspection as a major risk factor for *T. solium* transmission to humans in Zambia [11]. This group of people therefore serves as a critical point in the epidemiology of *T. solium* transmission in the study area. Knowledge plays a critical role in the prevention and control of diseases [37]. In studies on health education intervention conducted in Mexico and Southern Tanzania, it was shown that knowledge of *T. solium* infection played an important role in preventing transmission in the human-pig cycle [38, 39]. The poor knowledge of *T. solium* transmission among the respondents in this study was evident in their poor practices including defaecation on open fields and poor hand washing. As reported, infection with *T. solium* is important in areas with low socio-economic development, poor and inadequate sanitary facilities, and where pigs run loose scavenging for food, and with a ready access to human faecal material [40].

In Africa in general, animals including pigs are allowed to roam about freely as a result of inadequate feed and also to enable the animals utilize waste products often disposed poorly and convert such to meat [41]. A previously established report showed that there was a significant association between porcine cysticercosis and accessibility of pigs to human faecal material [42]. Therefore, this practice of defaecation on open fields and farmlands by some of the respondents in this study is an important point to consider when investigating the epidemiology of *T. solium* infection. Furthermore, the larger proportions (67.5%) of the respondents indicated knowing somebody suffering from epilepsy, a major manifestation of neurocysticercosis caused by *T. solium*. This corroborates the earlier report which indicated high prevalence of epilepsy in the country [43]. Likewise, it should be noted that pork consumption has been found to be a significant risk factor associated with neurocysticercosis among epileptic patients in a study in northern Tanzania [44]. Unfortunately however, none of the respondents in this present study knew that the *T. solium* in pigs could cause epilepsy despite the poor practices related to its transmission. Worse still, cases of epilepsy which are common particularly in the rural settings are often attributed to some fetish power at work [45]. From the small number of community-based studies available in the country, the point prevalence of epilepsy varies from 5.3 to 37 per 1 000 [18, 23, 43]. This notwithstanding, there is lack of functional primary health care facilities in such rural settings and as a result, the victims are often managed using traditional approach. Overall, epilepsy places particular demands on health, dramatically increasing the burden of disease given the social stigmatization and discrimination [46]. In the same vein, the study observed that only lower proportions of the respondents engaged in regular and periodic deworming. Our finding is similar to the report which indicated that the farmers in Soroti, Uganda had no regular strategies to control worms amongst themselves or their pigs [47]. This is a matter of public health concern considering the practice of defaecation on open fields and the fact that a single carrier of the worm if untreated and the worm remains active will continue to shed millions of eggs into the environment through defaecation for up to a period of 30 years [48]. Further to this observation, the entire respondents (100%) did not know that lack of regular deworming as well as improper washing of hands after using the toilet could facilitate *T. solium* transmission to man. This portends a high risk of *T. solium* infection transmission in the study setting and future research is required to screen these pig workers/consumers and others in similar settings for taeniid eggs.

## Conclusion

This study reveals a 4.4% prevalence of porcine cysticercosis among slaughtered pigs in Ibadan, South-Western Nigeria. It also reports associated increased odds of poor knowledge and practices related to *Taenia solium* transmission among the pig workers/consumers in the study area, reportedly known for high prevalence of epilepsy. It further shows that the respondents purchasing pork from home slaughter and those lacking toilet facility were less likely to have good knowledge and practices related to *T. solium* transmission to humans. These groups therefore serve as a critical factor in the epidemiology of *T. solium* infection in the area. The age (p = 0.000) and sex (p = 0.000) of the respondents as well as duration spent (p = 0.003) were other related significant factors. Since such findings might not be limited to the study area alone, but also characteristic of most developing sub-Saharan countries, there is a need for synergy between relevant local and international stakeholders towards promoting Public Health awareness campaigns about *Taenia solium* infection particularly among the occupationally exposed groups. In addition, stakeholders in the pig production industry should ensure regular deworming programmes and limit free ranging of pigs in order to curtail possible transmission of the parasite to man. Furthermore, a continuous systematic surveillance strategy for *Taenia solium* in pigs among farms in Nigeria and other developing countries at large will go a long way to stem the prevalence of this parasite and its associated public health implications.

### What is known about this topic

- That porcine cysticercosis is endemic in Nigeria;
- That humans could be exposed to *Taenia solium* infection.

### What this study adds

- It determines the current prevalence of porcine cysticercosis in slaughtered pigs in Ibadan, south-western Nigeria;
- It revealed that the current knowledge and practices related to porcine cysticercosis among pig handlers/consumers are poor and may enhance *Taenia solium* transmission to humans; thus making these occupationally exposed people a critical group to be considered in the epidemiology of *Taenia solium* transmission in the area;
- It indicated purchasing pork from home slaughter as well as lack of toilet facilities as exposure risk factors to *T. solium*/ infection in humans.

## Competing interests

The authors declare no competing interests.
